# Systemic Injection of Kainic Acid Differently Affects LTP Magnitude Depending on its Epileptogenic Efficiency

**DOI:** 10.1371/journal.pone.0048128

**Published:** 2012-10-31

**Authors:** Luz M. Suárez, Elena Cid, Beatriz Gal, Marion Inostroza, Jorge R. Brotons-Mas, Daniel Gómez-Domínguez, Liset Menéndez de la Prida, José M. Solís

**Affiliations:** 1 Hospital Universitario Ramón y Cajal, IRYCIS, Madrid, Spain; 2 Instituto Cajal CSIC, Madrid, Spain; 3 Universidad Europea de Madrid, Madrid, Spain; 4 Universidad de Chile, Santiago, Chile; University of Pittsburgh, United States of America

## Abstract

Seizures have profound impact on synaptic function and plasticity. While kainic acid is a popular method to induce seizures and to potentially affect synaptic plasticity, it can also produce physiological-like oscillations and trigger some forms of long-term potentiation (LTP). Here, we examine whether induction of LTP is altered in hippocampal slices prepared from rats with different sensitivity to develop *status epilepticus* (SE) by systemic injection of kainic acid. Rats were treated with multiple low doses of kainic acid (5 mg/kg; i.p.) to develop SE in a majority of animals (72–85% rats). A group of rats were resistant to develop SE (15–28%) after several accumulated doses. Animals were subsequently tested using chronic recordings and object recognition tasks before brain slices were prepared for histological studies and to examine basic features of hippocampal synaptic function and plasticity, including input/output curves, paired-pulse facilitation and theta-burst induced LTP. Consistent with previous reports in kindling and pilocapine models, LTP was reduced in rats that developed SE after kainic acid injection. These animals exhibited signs of hippocampal sclerosis and developed spontaneous seizures. In contrast, resistant rats did not become epileptic and had no signs of cell loss and mossy fiber sprouting. In slices from resistant rats, theta-burst stimulation induced LTP of higher magnitude when compared with control and epileptic rats. Variations on LTP magnitude correlate with animals’ performance in a hippocampal-dependent spatial memory task. Our results suggest dissociable long-term effects of treatment with kainic acid on synaptic function and plasticity depending on its epileptogenic efficiency.

## Introduction

Seizures have profound physiological and neurological *sequelae*. Recurrent seizures and *status epilepticus* presumably trigger an initial cascade of events including kinase activation, oxidative stress, neuronal damage and glial activation that change cellular and synaptic function [1]. In the particular case of synaptic function, seizures affect cellular processes accompanying a form of plasticity, namely long-term potentiation (LTP). LTP involves persistent changes of synaptic efficacy and is proposed to capitalize cellular processes required for learning [2]. Electrophysiological studies in animal models of epilepsy show that repeated seizures have deleterious consequences on LTP [3], [4]. Such effects have been proposed to be linked to the saturation of synaptic responses or due to impairment of LTP-associated molecular mechanisms caused by epileptiform bursts of activity [5]–[8]. Seizure-induced saturation of cellular resources available for plasticity would in turn potentially affect memory function [9]–[11].

To understand the molecular mechanisms underlying these changes, several animal models have been developed, with kindling stimulation and systemic injection of pilocarpine or kainic acid being the most popular methods. Repeated systemic injections of low doses of kainic acid, a glutamate receptor agonist, has revealed as good strategy to model the clinical and neuropathogical features of temporal lobe epilepsy (TLE) with reduced mortality [12], [13]. In vitro, kainate produces both physiological-like gamma oscillations and increases of excitability resulting in epileptiform activity. Presumably, low concentration of kainate presynaptically favour GABAergic release resulting in the rhythmic entrainment of pyramidal cell firing giving rise to local field potential gamma oscillations [14], [15]. At higher concentrations, increases of excitability dominate network dynamics provoking seizure-like events [15]. Intriguingly, recent evidence suggests that the ability of a circuitry to produce gamma activity is inversely related with its intrinsic epileptogenicity [16]. Given that kainate can directly regulate receptor trafficking and synaptic plasticity [17], [18], it is therefore possible that the epileptogenic efficiency of kainate directly interfere with plasticity function in TLE models.

In the present article, we determined whether the induction of LTP by theta-burst stimulation was altered in hippocampal slices obtained from rats treated with multiple, low-doses of intraperitonal injections of kainic acid in order to develop *status epilepticus* and chronic epilepsy. Consistent with previous findings in kindling models, LTP magnitude was reduced in epileptic rats when tested in the chronic phase. Interestingly, we found a group of rats resistant to develop *status* even after several accumulated doses of kainate. Resistant rats did not become epileptic and exhibited no signs of hippocampal sclerosis, i.e. neuronal loss and mossy fibre sprouting. In these animals, theta-burst stimulation induced a LTP of higher magnitude than that obtained from control rats. We found mild but significant positive correlation between the magnitude of LTP and the animal’s performance only in the hippocampal-dependent version of an object recognition task which also included evaluation of recognition memory. These findings indicate that kainic acid injections differentially modulate synaptic function depending on its epileptogenic efficiency.

## Materials and Methods

All procedures met the European guidelines for animal experiments (86/609/ECC). Protocols were approved by “Comité Ético de Experimentación Animal del Instituto Cajal” and “Comité Ético de Bienestar Animal” at Hospital Ramón y Cajal (animal facilities ES280790002001) for application grants MemStick (201600), BFU2009-07989 and PIU081067.

### Animals

Adult male Wistar and Sprague-Dawley rats weighing 180–200 g (45–50 days) were obtained both from the Harlan Laboratories and from our animal facilities (Instituto Cajal; Hospital Ramón y Cajal). Rats were i.p. injected with kainic acid (5 mg/kg) at hourly intervals until they reached the *status epilepticus* [12]. Seizures were scored according to Racine [19] (see first section of Results). The *status* was defined as a continuous convulsive condition lasting longer than 30 min. Diazepam (4 mg/kg, i.p.) was injected 1 hour after and repeated during the following 24 hours if convulsive behaviour persisted. To reduce mortality post-*status*, rats were i.p. injected with 2.5 ml 5% dextrose several times a day, and diet was supplemented with fruit and powder milk during the following 2–3 days. The control group was composed of rats treated with vehicle (saline) instead of kainic acid and received similar treatment than the experimental group. The observations described in this paper regarding different sensitivity of animals to systemic kainate injections were obtained from a large cohort of rats under the framework of different research projects in the lab of LMP (96 Wistar rats and 57 Sprague-Dawley animals). The present study was carried out in subgroups depending on the particular experimental design and the laboratory working plan. The number of animals and the sampling criteria are specified for each experimental procedure.

### Object Recognition Task

We checked for hippocampal-dependent spatial memory and novel object recognition memory using an object recognition task [20] between 6–8 weeks post-injection. Rats were randomly assigned to one of three groups in each strain: a) Wistar control n = 11, resistant n = 4, epileptic n = 13; b) Sprague-Dawley control n = 14, resistant n = 5, epileptic n = 12. Importantly, behavioral tests were suspended for at least two hours for those rats exhibiting spontaneous seizures. We also considered possible pre-seizure effects by excluding from the analysis those animals experiencing at least one seizure within the next hour after completing the test. Reactions to spatial changes and novel objects were evaluated by the exploration of objects placed in an open field (rectangular: 50×56 cm×83 cm). Animals had access to room visual cues during the whole experiment. Black curtains surrounded the field and a ceiling video camera was used to monitor rats behavior for offline analysis using a computer tracking system (Ethovision 1.90, Noldus IT). All objects were heavy enough not to be displaced by the rats and pilot experiments confirmed animals did not have preferences for them. Rats were habituated to the open field 4 times once a day (15 min free exploration) over 4 consecutive days. Immediately after habituation, animals were tested in five sessions (3 min each) separated by 5 min interval and grouped in three different phases: familiarization, spatial change and novel object recognition. During the familiarization phase (two trials) five objects were simultaneously placed in the open field. In the spatial change phase (two trials) two objects were displaced. In the novel object recognition phase (1 trial), a new object was substituted for the upper-left object. After each trial, the field and the objects were cleaned with acetic acid (0.1%) to remove odor cues. Exploration was evaluated by the time spent in active contact with the objects (e.g. sniffing). A contact was defined as the rat’s snout touching an object or directing at least 1 cm to it. Reaction to spatial changes was assessed by the comparison between the time spent on exploring the displaced objects during the familiarization phase and the time spent on exploring the same objects during the spatial change phase. Reactions to novel objects were assessed by comparing average time spent on exploring the familiar objects (never moved objects) versus that spent on exploring the new object. Chance level was thus defined at zero.

### Chronic Electrophysiology

Some rats were implanted for electrographic depth recordings from the dorsal hippocampus typically 6–8 weeks post-injection: n = 6 control, n = 4 resistant and n = 6 epileptic. For electrode implantation, animals were anesthetized with isofluorane (1.5–2%) in oxygen (30%) and continuously monitored with an oximeter (MouseOX, Starr Life Sci). Local field potential recordings were obtained either from 50 µm nichrome/formvar wires (impedance 0.3–0.5 MΩ) or 16-channel silicon probes (NeuroNexus Tech; 0.7–1.2 MΩ). Implantation coordinates were between 3.9–4.8 mm posterior to bregma and 3 mm from the midline. We typically adjusted the implantation depth guided by intra-operative electrophysiological recordings. Two screws served as a reference and ground at the occipital region. After recovering from surgery, hippocampal activity was monitored during several behavioral conditions (walking, running, immobility, sleep). Electroencephalographic recordings were performed daily, between 8 a.m. and 7 p.m. over the course of up to 2–3 weeks per rat. During these sessions, animals were submitted to several behavioral tasks (including object-recognition tasks) and monitorization of basal activity during large periods of spontaneous exploration, immobility and sleep. Animals were also continuously video-taped in periods of 48–72 hours for seizure detection. Spontaneous seizures were classified according to clinical signs using the Racine scale [19]. Electrographic signals were pre-amplified using field-effect transistors and further amplified and digitized at different sampling rates. For offline analyses, recordings were band-pass FIR filtered between 1 and 5000 Hz and down-sampled at 4800 Hz. Electrographic seizures were identified as a typical ictal pattern using the following criteria: a) discharge amplitude larger than 2.5SD baseline and b) continuous (>20 sec) large power spectrum in the 5–20 Hz band. Potential seizure events were double checked for the typical appearance of rhythmic spike-and-wave discharges. After completing recordings brains were fixed in 4% paraformaldehyde for electrode placement verification. Wire electrodes were found in the dorsal CA1 region typically below the pyramidal layer at sites within the stratum radiatum or near to the stratum lacunosum moleculare. With 16-channel silicon probes, LFP signals were selected from electrodes at equivalent strata to match similar recording sites using wires.

### Slice Preparation

Slices were prepared from a group of epileptic (Wistar n = 7, Sprague-Dawley n = 6), resistant (Wistar n = 2, Sprague-Dawley n = 8) and control (Wistar n = 4; Sprague-Dawley n = 7) rats, after completing behavioural tasks (>10–12 weeks post-injection). Different number of slices was used for input-output and plasticity studies. No more than 2 slices per rat were included for each type of experiments. Rats were decapitated after applying isoflurane anesthesia, and the brain was quickly removed and dropped into chilled Krebs-Ringer bicarbonate (KRB) solution containing (in mM): 119 NaCl, 26.2 NaHCO_3_, 2.5 KCl, 1 KH_2_PO_4_, 1.3 MgSO_4_, 2.5 CaCl_2_ and 11 glucose, gassed with 95% O_2_ and 5% CO_2_. Transverse dorsal hippocampal slices (400 µm) were obtained using a vibratome (Pelco 101) and then placed in a holding chamber for at least 3 h at room temperature (20–25°C) following standard methods. A single slice was transferred to a submersion-type recording chamber continuously perfused (1.9–2 ml/min) with standard KRB solution. Experiments were carried out at 31–32°C.

### *In vitro* Electrophysiology

Evoked field excitatory postsynaptic potentials (fEPSP) and presynaptic fiber volleys (FV) were recorded in CA1 stratum radiatum with tungsten microelectrodes (1 MΩ) connected to an AI-401 preamplifier (Axon Instruments, Foster City, CA) plugged to a CyberAmp 320 signal conditioner (Axon Instruments). These field responses were evoked every 15 seconds by stimulating Schaffer collateral (SC)-commisural fibers with biphasic constant-current pulses (20–40 µA; 100 µs per polarity; 0.066 Hz) delivered through bipolar tungsten insulated microelectrodes (0.5 MΩ) at CA1 mid-stratum radiatum. Stimulus strength was adjusted to evoke a fEPSP approximately half of its maximal amplitude. Electrical pulses were supplied by a pulse generator A.M.P.I. Mod. Master 8 (Jerusalem, Israel) connected to a biphasic stimulus isolator unit (Cibertec, Madrid, Spain). After a baseline period of at least 20 min, LTP was elicited with theta burst stimulation (TBS) consisting of 10 trains of four pulses at 100 Hz separated by 200 ms.

Evoked responses were digitized at 20 kHz using a Digidata 1200AE-BD board (Axon Instruments), and stored on a personal computer running Windows™ and using pCLAMP 8.0.2 software (Axon Instruments). The synaptic strength was calculated using the initial rising slope phase of the fEPSP to avoid contamination of the response by the population spike. We also used pCLAMP-8.0.2 software for these calculations. Data were normalized with respect to the mean values of the responses at the last 20 min of baseline period in standard medium.

### Histology: Immunohistochemistry and Timm Staining

Upon completion of behavioral and chronic electrophysiological experiments (>10–12 weeks post-injection), some rats were perfused intracardially with 30 ml of phosphate buffered saline 0.1 M, pH = 7.3 (PBS), 0.2% heparin, followed by 200 ml of 4% paraformaldehyde in PBS. See Results for details on the number of animals. Coronal sections of 100 or 50 µm were cut on a vibratome. For immunostaining, free-floating sections from levels between −5.0 mm to −5.5 mm from bregma were incubated in 1% H_2_O_2_ for 15 min. After PBS washing several times, sections were maintained during 1 hour in PBS containing 10% fetal bovine serum (FBS) and 0.25% Triton and then incubated overnight at 4°C in monoclonal anti-NeuN antibody (1∶1000, Bachem) diluted in PBS containing 1% FBS and 0.25% Triton. On the second day, sections were washed and incubated for 2 hours in biotinylated secondary antibody (anti-mouse IgG, 1∶200, Jackson) and for 1 hr in avidin-biotin- peroxidase complex (1∶1000, Vector) diluted in PBS-1% FBS. Sections revealed with 0.05% 3,3-diaminobenzidine and 0.01% H_2_O_2_, and mounted on slides coverslipped with glycerol and Eukitt (Fluka). We also validated the presence of mossy fiber sprouting (MFS) using the Timm staining in coronal slices from levels −5.5 mm to about −6 mm from bregma. For Timm staining, rats were perfused with Na_2_S 0.1% in PBS 0.1 M (heparin 0.2%) at 4°C before fixation. Free-floating sections (70 µm) were developed in dark, using arabig gumm, citric acid, hydroquinone and silver nitrate. Electrode verification from animals chronically implanted were performed using coronal sections (100 µm) and the thionin staining.

### Statistical Analysis

Data are expressed as mean ± SEM. Statistical analyses were performed using SPSS 18.0 for Windows. Mean values of fEPSP slope or FV amplitude given throughout the text correspond to averages of 5 min periods. Statistical differences were assessed by one- or two-way analysis of variance (ANOVA) followed by Bonferroni t-test or by two-tailed unpaired Student’s t-tests with P<0.05 for statistically significance. To evaluate discrimination ratios from the object recognition task we used one-way ANOVA that considered group (control, resistant and epileptic) and strain factors. Discrimination ratios for each group were also compared with chance level performance (zero) using one-sample t-tests. To test for specific correlations between LTP magnitude and behavioral variables, the Pearson’s correlation analysis was applied.

## Results

### Effect of Systemic Injection of Kainate: Epileptic and Resistant Phenotypes

**Figure 1:**
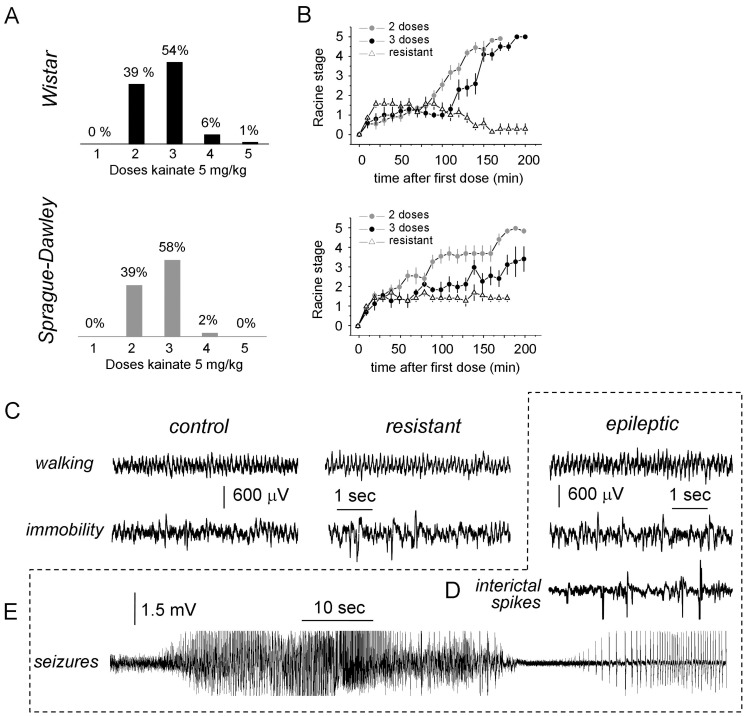
Effect of systemic injections of kainate in Wistar and Sprague-Dawley rats. (A) Multiple doses of 5 mg/kg kainate were used to induce *status epilepticus* (SE) in adult male rats. The histograms show percentage of rats exhibiting SE in Wistar (n = 96) and Sprague-Dawley animals (n = 57). No differences were found between strains. (B) Progression to SE in Wistar and Sprague-Dawley rats as evaluated with the Racine scale. Data from for n = 10 rats entering SE with 2 and 3 doses in each strain. A group of rats were resistant to develop SE (n = 10 Wistar and n = 7 Sprague-Dawley). (C) Chronic electroencephalographic (EEG) recordings were obtained from the dorsal hippocampus in a group of animals. Representative examples of EEG recording during walking and awake immobility are shown for control, resistant and epileptic animals. (D,E) Epileptic rats also exhibited epileptiform events like interictal spikes (D) and spontaneous seizures (E). The discontinous line box group data obtained from the same epileptic rat.

Using multiple low doses (5 mg/kg) of kainic acid resulted in the development of *status epilepticus* in a majority of both Wistar (n = 82 out of 96, 85%) and Sprague-Dawley rats (n = 41 out of 57, 72%). Rats required between 2–3 accumulated doses of kainate to enter *status* with no difference between strains (Fig. 1A). Progression to *status* was characterized by a consistent sequence of events [12], [19] including (Fig. 1B): a) orofacial automatisms (stage 1); b) head nodding (stage 2); c) forelimb clonus (stage 3); d) forelimb clonus with rearing (stage 4) and e) forelimb clonus with rearing and fallings (stage 5). Clear separation between stages 1 and 2 was difficult in the kainate model, which was typically characterized by the presence of wet-dog shakes (latency W 53±5 min; SD 67±9.3 min). Individually, rats required different accumulated doses to enter the *status* which was typically preceded by stage 3 and 4 seizures. *Status* took longer to start in Sprague-Dawley (188±12 min) than in Wistar rats (154±4 min; p = 0.0035), but was more abrupt in Wistar animals (Fig. 1B).

Rats that entered the *status* became epileptic when tested at least 6–8 weeks after treatment (Fig. 1C,E), typically exhibiting spontaneous seizures of grade 3–4 [13]. Chronic electrophysiological recordings from the dorsal hippocampus of these rats (n = 6) showed also the presence of abnormal forms of activity, including interictal spikes (Fig. 1D). These epileptiform patterns were recorded irregularly during some, but not all, episodes of slow-wave sleep and awake immobility. During most episodes of walking and behavioural immobility hippocampal EEG patterns appeared normal in epileptic rats, characterized by theta and large irregular activity, respectively (Fig. 1C).

A group of rats (Wistar n = 14 out of 96, 15%; Sprague-Dawley n = 16 out of 57; 28%) were resistant to develop *status,* rarely reaching Racine stages larger than 2 when injected with kainate (Fig. 1B). In some of these animals, we accumulated up to 6 doses of kainate 5 m/kg without any further clinical effect. Some resistant animals experienced brief seizing episodes of forelimb clonus during the induction phase but behaved apparently normally in between, i.e. they were reactive to stimulation; walked around, eat, etcetera. Spontaneous seizures were never observed in resistant rats when recorded after 6 weeks post-injection (n = 4 animals). Instead, resistant rats exhibited EEG patterns similar to control animals (n = 6) in comparable behavioural conditions: i.e. theta activity during waking and running and large irregular sharp-wave activity during immobility (Fig. 1C).

### Histological Changes in the Hippocampus of Kainate-injected Rats

**Figure 2:**
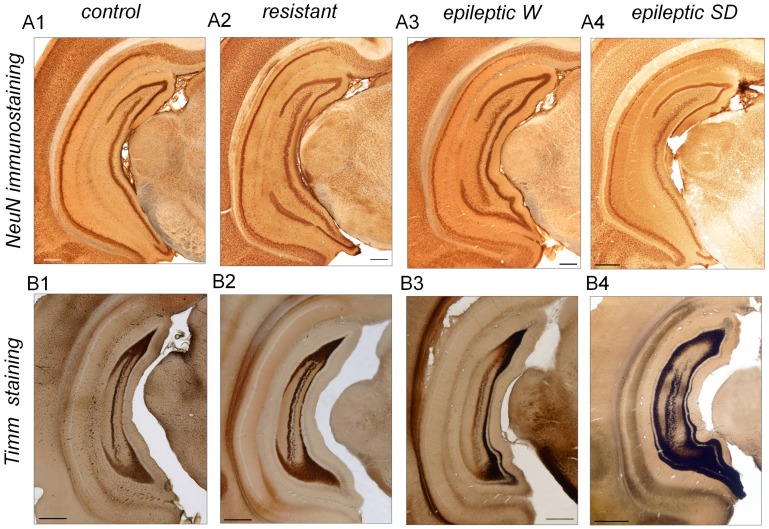
Histological studies. (A) NeuN immunostaining was used to evaluate cell loss in coronal slices from control (A1), resistant (A2) and epileptic (A3,A4) animals. No strain differences were apparent for control and resistant rats, while different degree of cell loss was evident in Sprague-Dawley (SD) epileptic rats as compared with Wistar (W) [13]. (B) Timm staining was used to evaluate mossy fiber sprouting (MFS). Again, no apparent changes were found in control (B1) and resistant animals (B2), while MSF was obvious in the ventral hippocampus of epileptic animals (B3,B4). Scale bars are 500 µm.

We used NeuN immunostaining and histological techniques to look at brain differences between epileptic (Wistar n = 10; Sprague-Dawley n = 7) and resistant rats (Wistar n = 3; Sprague-Dawley n = 4) when compared with control animals (Wistar n = 3; Sprague-Dawley n = 3; Fig. 2). Overall, immunostained sections from control and resistant rats were indistinguishable when blindly evaluated by two independent researchers with no apparent strain differences neither. However, obvious differences were observed for epileptic animals with Sprague-Dawley rats exhibiting larger damage than Wistar, as previously reported [13]. In epileptic rats, neuronal loss was detected both in the dorsal and the ventral hippocampus typically affecting CA1 and CA3 regions, together with cell layer dispersion in the dentate gyrus (Fig. 2A3, A4 versus A1). No apparent cell damage was observed in the hippocampus of resistant rats (Fig. 2A2 versus A1). We also checked for the presence of mossy fiber sprouting (MFS), a hallmark of TLE characterized by the sprouting of recurrent excitatory collaterals of granule cell axons, using Timm staining. Both control and resistant rats exhibited no signs of mossy fiber sprouting (Fig. 2B1 versus B2), which was present in the ventral hippocampus of epileptic rats (Fig. 2B3,B4 versus B1). Timm scores values of resistant rats (dorsal 0.38±0.16, ventral 1.8±0.28) were similar to those we reported previously for control animals [13]. Altogether, our electrophysiological and histological data suggest that rats resistant to kainate-induced *status* did not develop TLE in contrast to animals that entered the *status*, which became epileptic and had histological signs of hippocampal sclerosis.

### Basal Synaptic Transmission and Paired-pulse Facilitation in Kainate-injected Rats

**Figure 3:**
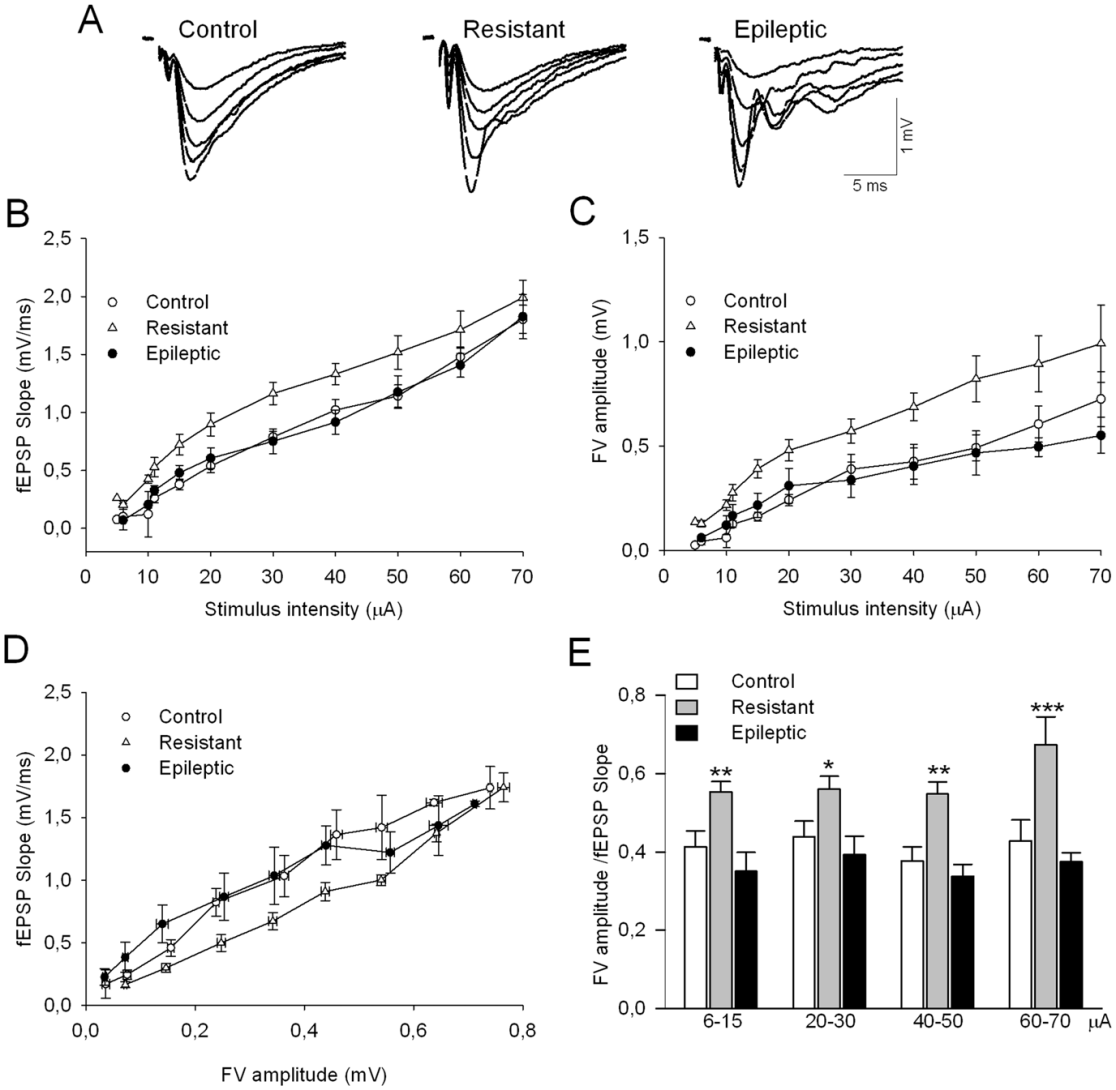
Basal synaptic transmission is altered after kainate treatment. (A) Superposition of fEPSPs (5 consecutive responses) evoked by various stimulus strengths in single representative experiments obtained from control, resistant and epileptic rat. Stimulus-response curves for fEPSP (B) and the fiber volley FV (C) obtained in slices from the control (16 slices from 11 rats), resistant (15 slices from 10 rats) and epileptic (16 slices from 13 rats) groups. (D) Input-output curves were created by comparing FV amplitudes with fEPSP slopes from data depicted in B and C. (E) FV/fEPSP ratios for different ranges of stimulus strength shown in D confirm that resistant rats present a reduction of synaptic efficacy when compared with control animals (*P<0.05).

We then tested for changes of CA1 synaptic function using slices prepared from resistant, epileptic and control rats. We first tested for basal synaptic transmission using input-output (I/O) curves to look at the relationship between slopes of the field excitatory postsynaptic potential (fEPSP) and the intensity of Schaffer collateral stimulation. A two-way ANOVA of these curves yielded a significant effect of group factor (F(2,281) = 25.54; P<0.001). Difference in slope values for group factor was greater than would be expected by chance after allowing for effects of differences in strain factor. We therefore pooled data from control (n = 16 slices from 11 rats), resistant (n = 15 slices from 10 rats) and epileptic rats (n = 16 slices from 13 animals) of the two strains (Fig. 3A,B). Post-hoc analysis revealed that I/O curve of resistant rats was statistically different from control group (P<0.05; Bonferroni t-test), whereas no significant differences were observed in I/O curve obtained in epileptic rats when compared with control animals (P>0.05; Bonferroni t-test). This upward shift of I/O curves in resistant rats suggests facilitated synaptic responses in this group.

We wonder whether larger synaptic responses in resistant rats were caused by enhanced pre-synaptic excitability of the Schaffer afferent pathway. To this purpose, we looked at the stimulus dependency of the fiber volley (FV) amplitude, which is proportional to the number of presynaptic axons recruited by stimulation (Fig. 3C). We also found an upward shift of FV amplitudes in resistant rats when compared with control and epileptic animals (P<0.0001; Bonferroni t-test), indicating that enhanced post-synaptic responses were at least partly reflecting larger fiber recruitment. In order to reliably determine whether the synaptic efficacy was modified in resistant rats we plotted fEPSP slopes against FV amplitudes (Fig. 3D). We observed a rightward shift in resistant rats respect to control and epileptic animals, suggesting that recruitment of equivalent number of fibers evoked lower responses in resistant rats. The statistical significance of this change was revealed when grouping FV/fEPSP ratios against stimulus intensities (Fig. 3E; P<0.05, Bonferroni t-test). These results indicate that although resistant rats presented an upward shift in I/O curves for both fEPSP and FV, the net result was a reduction of synaptic efficacy at CA3-CA1 synapses in these animals. Strikingly, epileptic rats showed similar I/O behaviour compared with control animals indicating maintained synaptic efficacy. Moreover, similarities of FV/fEPSP ratios and I/O curves in epileptic and control rats, suggest poor heterosynaptic contamination of SC-evoked responses by potential activation of the temporoammonic pathway in the sclerotic hippocampus.

**Figure 4:**
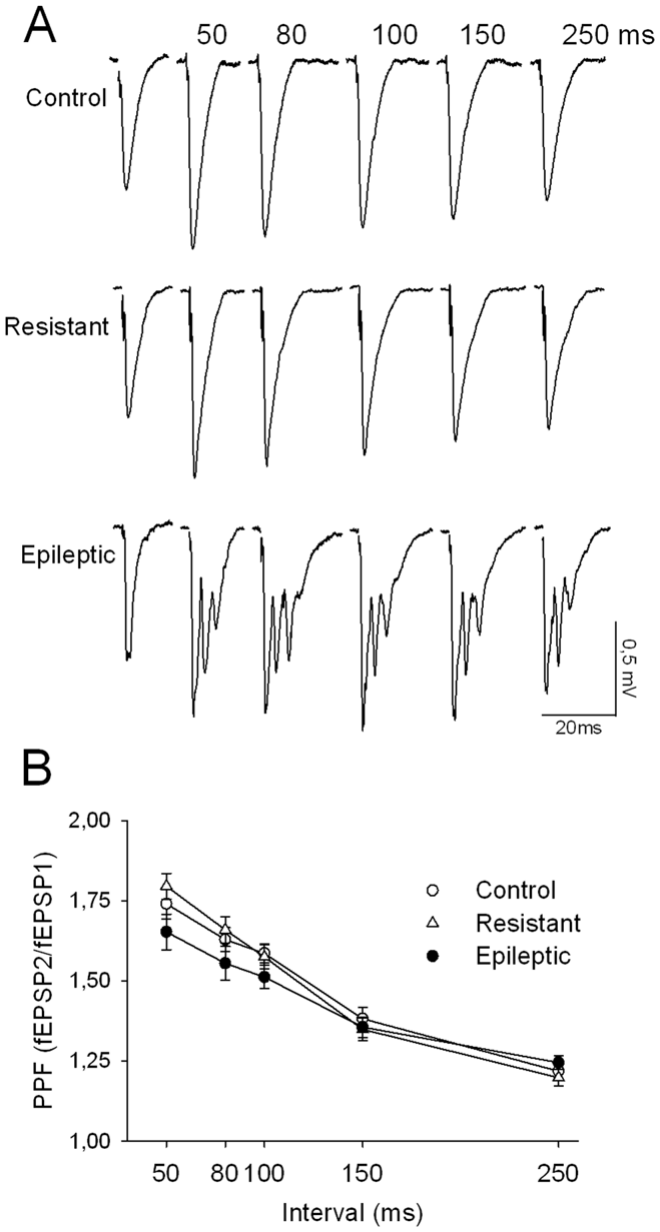
Presynaptic release probability, estimated from paired-pulse facilitation ratios, was modified neither in resistant nor epileptic rats. (A) Representative traces recorded at different inter-pulse intervals indicated by the numbers above the records. The first record in a row corresponds to fEPSPs evoked by the first pulses. (B) Summary data of paired-pulse facilitation ratios obtained in the same slices used to induce LTP in figure 5. No significant differences were found among experimental groups (P>0.05). Data from n = 16 slices from 11 control rats; n = 7 slices from 7 resistant rats and n = 16 slices from 10 epileptic rats.

We also examined paired-pulse facilitation (PPF) by measuring the ratio between the second and the first fEPSP slopes at different inter-stimulation intervals (50, 80, 100, 150 and 250 ms). This stimulation paradigm is widely used to detect changes in release probability [21]. Figure 4 shows that PPF ratios were indistinguishable in the three experimental groups (F(2,220) = 2.835; P = 0.061), suggesting that presynaptic release of glutamate was not permanently altered after kainate treatment. Remarkably, the second stimulation pulse evoked synaptic potentials with multiple propagated population spikes in slices from epileptic rats (Fig. 4A), as previously described [22].

### LTP Induction in Kainate-treated Rats

**Figure 5:**
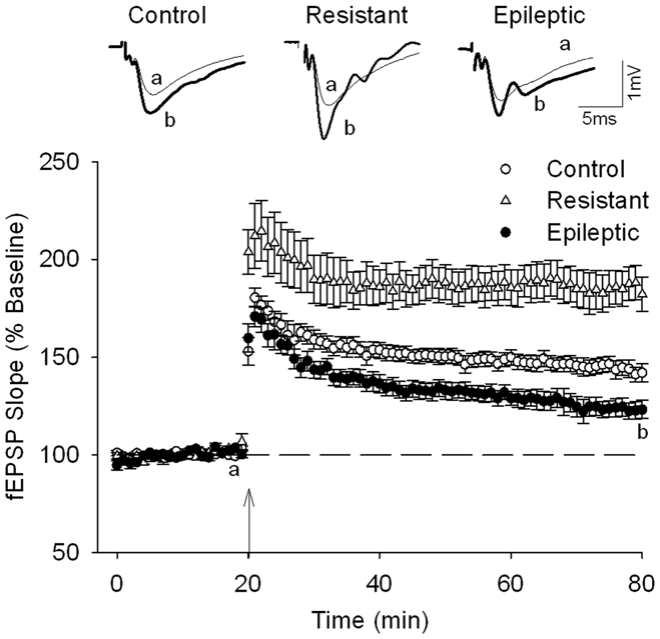
Theta-burst-induced LTP was enhanced in resistant rats but reduced in epileptic animals. Upper row shows representative averaged fEPSPs recorded at the time indicated by the letters on the graph to exemplify LTP induction in different experimental groups. Graphs plot summary data of experiments where theta burst tetanization was applied (indicated by the arrow) in control (n = 16 from 11 rats), resistant (n = 7 from 7 rats) and epileptic (n = 16 from 10 rats) slices.

*Status* induced by kindling stimulation or pilocarpine injection impairs LTP [3], [6], [8]. To examine whether this form of plasticity is also affected in the kainate model of TLE, we carried out a set of experiments where, after a baseline recording period of at least 20 min, trains of 10 theta bursts were applied to the Schaffer collateral pathway to evoke LTP (Fig. 5). Induction of LTP in slices from epileptic rats resulted in a smaller synaptic potentiation than that obtained in control slices (n = 16 slices; 145±4% from n = 11 control rats, vs. 124±4% in n = 16 slices from n = 10 epileptic rats, at 1 h after TBS; P<0.001; Students t-test), which is in accordance with deficits of LTP found in other experimental models of TLE. Unexpectedly, in slices from resistant rats (n = 7 slices from 7 rats) we found that TBS stimulation induced a robust and higher LTP (185±9% at 1 h after TBS) than that obtained in control animals (P<0.001; Students t-test).

#### Spatial memory function correlates with LTP magnitude

**Figure 6:**
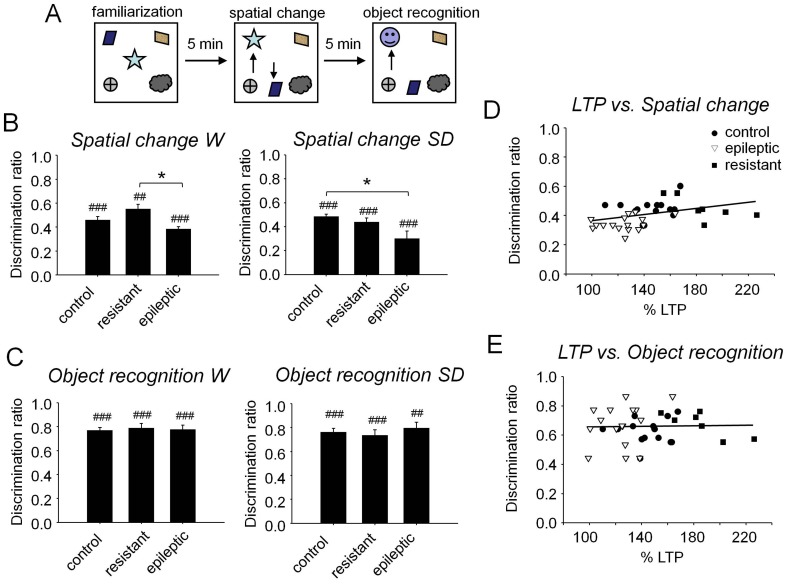
Relationships between LTP magnitude and memory function. (A) Animals were tested in five sessions (3 min each) separated by 5 min interval and grouped in three different phases: familiarization, spatial change and novel object recognition. During the familiarization phase (two trials) five objects were simultaneously placed in the open field. In the spatial change phase (two trials) two objects were displaced (arrows). In the novel object recognition phase (1 trial), a new object was substituted for the upper-left object (arrow). (B) Discrimination ratios for the spatial change tasks for Wistar (W) and Sprague-Dawley (SD) rats. * P<0.05 for pair-wise comparisons between groups; ^##^ P<0.01; ^###^ P<0.001 for comparisons with chance level. Note poor performance of epileptic rats in Sprague-Dawley (n = 12) but not in Wistar animals (n = 13). The control grop is composed of n = 11 Wistar and n = 12 Spague-Dawley rats. Resistant rats are n = 4 Wistar and n = 5 Spraque-Dawley. (C) Discrimination ratios for the novel object recognition. Note that deficits specifically affect hippocampal-dependent spatial memory and not recognition memory. (D) Positive correlation between LTP magnitude and discrimination ratios in the spatial memory task (r^2^ = 0.14, P = 0.017). (E) No correlation was found between LTP magnitude and discrimination ratios in the novel object recognition task.

We next wondered whether difference in the magnitude of LTP correlate with animal’s performance in a hippocampal-dependent memory task. To this purpose we had tested rats’ abilities in reacting to spatial changes using an object recognition task ([20]; Fig. 6A) well before preparing slices for LTP studies. Analysis of discrimination ratios to spatial change during the task showed a significant effect for groups in both strains [Wistar F(2,27) = 4.09, P<0.05; Sprague-Dawley F(2,30) = 3.47, P<0.05]. Differences between epileptic and control rats reached significance for Sprague-Dawley (P<0.05) but not for Wistar animals (Fig. 6B). Importantly, one-sample t-test revealed that discrimination ratios were significantly above chance level for all groups both in Wistar (control: t(10) = 12.35, P<0.001, resistant: t(3) = 13.34, P<0.01, epileptic: t(12) = 11.90, P<0.001) and Sprague-Dawley rats (control: t(13) = 18.38, P<0.001, resistant: t(4) = 12.27, P<0.001, epileptic: t(11) = 3.58, P<0.01), confirming that memory for spatial changes was preserved in the kainate model of TLE, in agreement with our previous findings using the Morris water maze [13].

We also controlled for hippocampal-independent memory abilities by testing the rat ability to recognize novel objects. Discrimination ratios showed no differences between groups for both strains [Wistar F(2,27) = 0.10, P = 0.90; Sprague Dawley F(2,30) = 0.45, P = 0.64] (Fig. 6C), with all ratios above chance level (Wistar control: t(10) = 24.76, P<0.001, resistant: t(3) = 19.30, P<0.001, epileptic: t(12) = 12.17, P<0.001; Sprague Dawley control: t(13) = 18.10, P<0.001, resistant: t(4) = 15.27, P<0.001, epileptic: t(11) = 12.52, P<0.001 ), revealing that all rats were similarly competent to recognize a novel object.

We next checked for relationship between the magnitude of LTP and the animal’s ability to discriminate for spatial changes. We found a mild positive correlation between the percentage of synaptic potentiation and the discrimination ratio in the spatial change task (r^2^ = 0.14, P = 0.017; Figure 6D). This interaction was also significant when considering only data from control and epileptic rats (r^2^ = 0.27, P = 0.003) or resistant and epileptic animals (r^2^ = 0.23, P = 0.021), but not for any group alone. These results indicate that part of the variation in animal’s performance could be explained by differences in available LTP resources. Remarkably, no correlation was found between synaptic potentiation and the discrimination ratio in the novel object recognition task (r = 0.02, P = 0.90; Fig. 6E), confirming that potential effects of plasticity changes are hippocampal-dependent.

## Discussion

In the present study, we show that LTP induction was impaired in hippocampal slices obtained from rats rendered epileptic after kainate-induced *status*. Notably, we found a group of rats resistant to develop *status* even after several accumulated doses of kainate. Slices prepared from these animals exhibited larger LTP magnitudes compared with control. We also report a mild but significant correlation between the magnitude of LTP and the animal’s performance in a hippocampal-dependent spatial memory task, suggesting that kainic acid differently modulate cellular processes involved in synaptic plasticity depending on its epileptogenic efficiency.

With the experimental procedure of multiple injections of a low-dose of kainate (5 mg/kg) *status epilepticus* is usually attained between two and three doses at high incidence but drastically reduced mortality rate [12]. Kainate-resistant rats tolerate accumulated doses of kainate without developing recurrent convulsive seizures and *status*. This group represents about 20% of our kainate-treated animals. A similar rate of resistant rats has been found with the lithium-pilocarpine model [23]. However, such a percentage contrasts with the success obtained by others using kainate [12] which reported that nearly all treated animals developed *status epilepticus*.

Data indicate that genetic and circuit factors interfere with the expression of epileptic phenotypes [24]–[28]. However, it is unclear why some rats are protected against the epileptogenic effects of kainate while others are sensible to develop recurrent seizures. One possibility is that in our hands the effective dose of kainate attained in the brain of resistant rats was lower than in the case of rats entering the *status*. This seems unlikely, because some of these animals displayed brief convulsive seizures although they did not develop *status*. Another possibility is that the initial kainate injections cause a post-translational modification of kainate receptors by SUMOylation or phosphorylation which promotes the internalization of these receptors in some animals [18]. This would render these rats potentially resistant to develop *status epilepticus* with subsequent kainate injections. Indeed, resistant rats share similar clinical signs with animals entering the *status* over the first 10 minutes after kainte injection (Fig. 1B). Such a process of epileptic tolerance has been observed with preconditioning seizures evoked by kainate injections [29]. Resistance to kainate-induced epilepsy has been also observed in immature rats where multiple kainate injections did not cause major hippocampal damage or electrographic seizures [30]. Inhibition of BDNF synthesis renders hippocampus of immature rats sensitive to damage caused by kainate injections [31]. Nevertheless, the role of BDNF on epilepsy is controversial, because both antiepileptic [32] and pro-epileptic effects have been reported [33]. Another possibility might be attributable to genotype differences among rats. For example, differences of subunit composition of kainate receptors might account for altered sensitivity to develop *status*. Polymorphisms in kainate receptors have been associated with response to anti-depressants [34] and experimental transgenic strategies suggest different seizure susceptibility for GluR5- and GluR6-containing receptors [14], [35]. Whether resistant rats have different genetic background that renders them less sensible to kainate injection remain unclear to us.

We did not find differences between control and epileptic rats when comparing their input/output curves indicating that basal synaptic transmission is not altered after an episode of *status epilepticus*, in agreement with in vivo data [22]. Even though, we detected that the magnitude of TBS-induced LTP was reduced in epileptic rats. This result is similar to that obtained with electrographic seizures [36], [37], pharmacological induced epileptiform activity [4], [38] and other experimental models such as kindling [3], [6], [39] and pilocarpine injections [23]. In general, impairment of this form of synaptic plasticity has been attributed to seizure related alterations of signalling cascades involved in the modulation of synaptic strength [2], [4], which would occlude or saturate subsequent generation of LTP by electrical stimulation. We think that our results are not consistent with this proposal because if such seizure-induced synaptic potentiation had taken place in our epileptic rats, we should have detected an increase of basal synaptic transmission in these animals. Other authors have argued against saturation mechanisms to explain kindling-induced impairment of LTP [6]. Interestingly, in hippocampal slices it has been shown that brief application of domoate, a kainate receptor agonist, causes a long-lasting potentiation of fEPSP which occludes further LTP induction by electrical tetanization, whereas prolonged application, which does not evoked synaptic potentiation, dramatically reduces subsequent induction of LTP [40]. This last situation might have occurred in our epileptic rats. We therefore propose that LTP deficits observed in these animals are more compatible with a failure of LTP induction mechanisms caused by the *status* than with saturation of synaptic potentiation.

Importantly, we have detected an enhancement of LTP magnitude in the group of rats where kainate administration did not generate *status*. These synaptic changes resemble those found in hippocampal CA1 area one day after lithium-pilocarpine injection although 6 weeks later these animals presented LTP impairment [23]. In our study, the increment of LTP magnitude in resistant rats was observed several weeks after kainate treatment, disclosing, thereafter, that these changes were due to the presence of kainate during LTP induction.

Persistent changes of synaptic efficacy are considered a cellular substrate underlying learning and memory processes. LTP is proposed to capitalize those cellular resources and it is used as a marker of cognitive abilities [41], [42]. Consistently, we found positive correlation between LTP and performance in a hippocampal-dependent spatial memory task. Possibly, in resistant animals kainate-activated receptors might undergo metaplastic alterations facilitating LTP expression and potentially impacting learning capabilities. Further work is required to better target the underlying mechanisms involved in this seizure resistant phenotype.
